# RNA secondary structure prediction with pseudoknots: Contribution of algorithm versus energy model

**DOI:** 10.1371/journal.pone.0194583

**Published:** 2018-04-05

**Authors:** Hosna Jabbari, Ian Wark, Carlo Montemagno

**Affiliations:** 1 Department of Computer Science, University of Vermont, Burlington, Vermont, United States of America; 2 Ingenuity Lab, Department of Chemical and Materials Engineering, University of Alberta, Edmonton, Alberta, Canada; 3 Southern Illinois University Carbondale, Carbondale, Illinois, United States of America; Bangladesh University of Engineering and Technology, BANGLADESH

## Abstract

**Motivation:**

RNA is a biopolymer with various applications inside the cell and in biotechnology. Structure of an RNA molecule mainly determines its function and is essential to guide nanostructure design. Since experimental structure determination is time-consuming and expensive, accurate computational prediction of RNA structure is of great importance. Prediction of RNA secondary structure is relatively simpler than its tertiary structure and provides information about its tertiary structure, therefore, RNA secondary structure prediction has received attention in the past decades. Numerous methods with different folding approaches have been developed for RNA secondary structure prediction. While methods for prediction of RNA pseudoknot-free structure (structures with no crossing base pairs) have greatly improved in terms of their accuracy, methods for prediction of RNA pseudoknotted secondary structure (structures with crossing base pairs) still have room for improvement. A long-standing question for improving the prediction accuracy of RNA pseudoknotted secondary structure is whether to focus on the prediction algorithm or the underlying energy model, as there is a trade-off on computational cost of the prediction algorithm versus the generality of the method.

**Results:**

The aim of this work is to argue when comparing different methods for RNA pseudoknotted structure prediction, the combination of algorithm and energy model should be considered and a method should not be considered superior or inferior to others if they do not use the same scoring model. We demonstrate that while the folding approach is important in structure prediction, it is not the only important factor in prediction accuracy of a given method as the underlying energy model is also as of great value. Therefore we encourage researchers to pay particular attention in comparing methods with different energy models.

## 1 Introduction

RNA molecules are crucial in different levels of cellular function, and their functions largely depend on their structures. RNA molecules are widely used as building blocks of nano-structures with various applications in biotechnology [1]. Since experimental methods for determining RNA structure are expensive, time-consuming and in some cases impossible, computational methods for prediction of RNA secondary structure (the set of base pairs) are valuable. The most widely used methods for prediction of RNA secondary structure, however, can deal only with pseudoknot-free structures (structures with no crossing base pairs), even though pseudoknots (structures with crossing base pairs) are known to be functionally important in many RNAs. Although several approaches have been proposed that predict pseudoknotted structures, it is still difficult to evaluate merits of each approach as they are different in their hypothesis and their underlying scoring function (i.e. energy model). In particular it is not known how much prediction accuracy depends on folding hypothesis (structure formation hypothesis) versus the underlying energy model. We aim to address this question by comparing performance of four RNA pseudoknotted secondary structure prediction methods with different folding hypotheses but the same energy model.

In this work, we thoroughly compare performance of four of the most general RNA pseudoknotted secondary structure prediction algorithms (IPknot [2], HotKnots [3], CCJ [4] and Iterative HFold [5]) with four different folding approaches (maximum expected accuracy, heuristic approach, minimum free energy and hierarchical folding hypotheses respectively) but the same underlying energy model (HotKnots V2.0 DP09) on a large dataset of known structures. All algorithms compared in this work are applicable to novel RNAs with unknown families; two are based on biologically sound folding hypotheses (CCJ and Iterative HFold), and all use an energy model that is, to the best of our knowledge, one of the best available energy models for prediction of pseudoknotted structure and is based on experimentally determined parameters. By comparing these algorithms with similar energy model, we provide a fair evaluation of advantages and disadvantages of each approach. In addition, we provide the first computational framework for the evaluation of two biologically sound RNA folding hypotheses (minimum free energy vs. hierarchical folding).

We selected these methods for our analysis for the following reasons: (1) they are all sequence-based (vs. alignment-based) methods and therefore, applicable to novel classes of RNAs including non-coding RNAs; (2) IPknot and HotKnots are two of the best performing algorithms in the comparison performed by Puton et al. [6]; CCJ and Iterative HFold are two of the newer methods for RNA pseudoknotted structure prediction and so were not part of Puton et al. comparison. The CCJ algorithm is the most general pseudoknot prediction algorithm that runs in *O*(*n*^5^) time and *O*(*n*^4^) space; with our implementation of the CCJ algorithm, we can predict structure of long (up to 500 length) RNA molecules. (3) all four methods use HotKnots V2.0 DP09 energy model (and parameters). We perform a thorough empirical evaluation of these methods on a large dataset of more than 600 structures, and present merits and shortcomings of each method.

## 2 Methods

**Fig 1:**
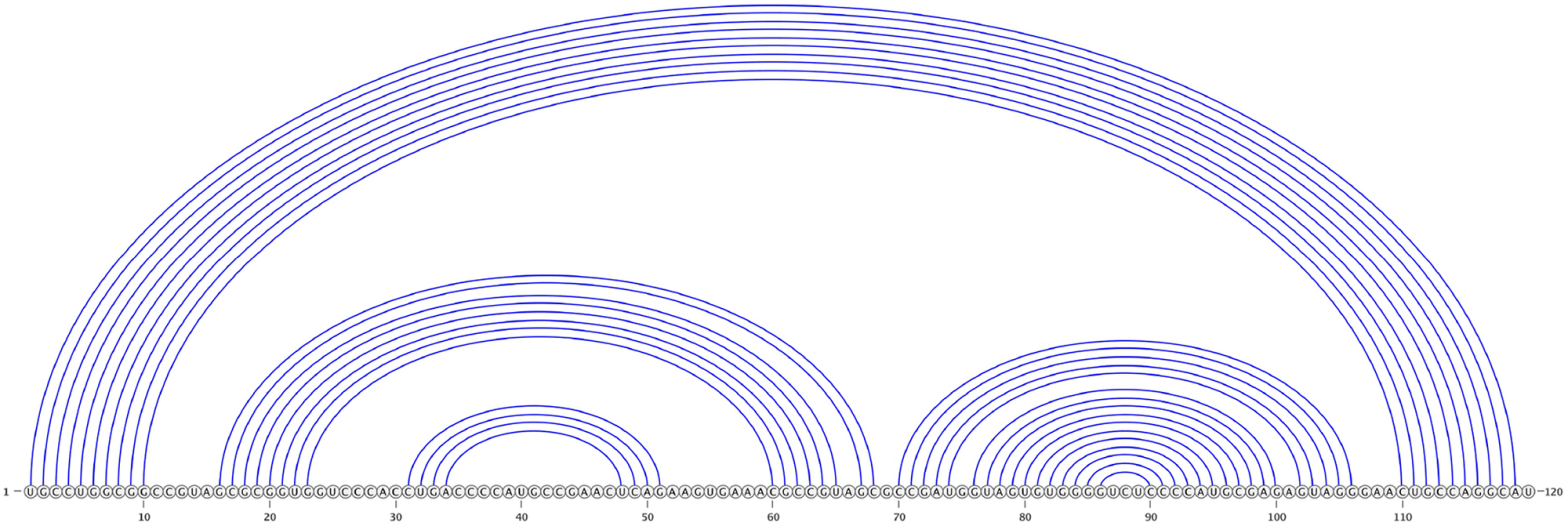
Pseudoknot-free secondary structure of 5S Ribosomal RNA, Escherichia Coli, PDB_00030 [9]. The horizontal line shows the backbone from 5’ to 3’ end, and arcs represent pairing between two nucleotides (bases). Note that there are no crossing arc in this molecule.

We represent an RNA molecule as a sequence of its four bases (A, C, G, and U) arranged on a line (representing the backbone) from 5’ (left) to 3’ (right) ends. *n* represents the length of the RNA molecule. Each base is referred to by its index. RNA secondary structure is then represented by a set of complimentary base pairs *i*.*j* (i.e., A.U, U.A, C.G, G.C, U.G and G.U). We represent base pairs by arcs connecting index *i* to index *j* (see Fig 1). There are different loop types based on the closing arc(s). Loops are differentiated based on their features. For a precise definition of loops we refer the readers to the work of Jabbari et al. [7] or Rastegari and Condon [8]. We refer to structures with no crossing arcs as *pseudoknot-free* and structures with crossing arcs as *pseudoknotted* structures.

### 2.1 Energy model

Energy of a secondary structure in energy-model based approaches used in this work is calculated as the sum of energies of its loops, as defined in the energy model. Here we use the DP09 parameter set of Andronescu et al. [3] used by the HotKnots V2.0 method. Table 1 summarizes values of the pseudoknotted parameters of this energy model.

**Table 1:** Energy parameters. This table provides the names, description and values of the energy parameters and functions used in this work. These parameters were derived for a temperature of 37°C and 1 M salt (NaCl) concentration. Values are reported in *kcal*/*mol*. DP09 column corresponds to the pseudoknotted parameters of HotKnots V2.0 [3], and T99 column corresponds to the standard Turner 99 parameters with Dirks and Pierce 2003 pseudoknotted parameter values [10].

Name	Description	DP09	T99
*P*_*s*_	exterior pseudoloop initiation penalty	−1.38	9.6
*P*_*sm*_	initiation penalty of pseudoloop inside a multiloop	10.07	15.00
*P*_*sp*_	initiation penalty of pseudoloop inside a pseudoloop	15.00	15.00
*P*_*b*_	initiation penalty of a band	2.46	0.2
*P*_*up*_	penalty for unpaired base of a pseudoloop	0.06	0.1
*P*_*ps*_	penalty for closed subregion inside a pseudoloop	0.96	0.1
*e*_*H*_(*i*, *j*)	energy of a hairpin loop closed by *i*.*j*		
*e*_*S*_(*i*, *j*)	energy of stacked pair closed by *i*.*j*		
*e*_*stP*_(*i*, *j*)	energy of stacked pair that spans a band	0.89 × *e*_*S*_(*i*, *j*)	0.83 × *e*_*S*_(*i*, *j*)
*e*_*int*_(*i*, *d*, *e*, *j*)	energy of a pk-free internal loop		
*e*_*intP*_(*i*, *d*, *e*, *j*)	energy of internal loop that spans a band	0.74 × *e*_*int*_(*i*, *d*, *e*, *j*)	0.83 × *e*_*int*_(*i*, *d*, *e*, *j*)
*a*	initiation penalty of an ordinary multiloop	3.39	3.4
*b*	multiloop base pair penalty	0.03	0.4
*c*	penalty for unpaired base of an ordinary multiloop	0.02	0
*a*′	initiation penalty of a multiloop that spans a band	3.41	3.4
*b*′	branch penalty in a multiloop that spans a band	0.56	0.4
*c*′	penalty for unpaired base in a multiloop that spans a band	0.12	0

### 2.2 Datasets

We use four datasets to analyze performance of our algorithms, namely HK-PK, HK-PK-free, IP-pk168 and DK-pk16. HK-PK (with 88 pseudoknotted structures) and HK-PK-free (with 337 pseudoknot-free structures) datasets were compiled from RNA STRAND database and were used as test datasets in evaluating HotKnots V2.0 [3]; IP-pk168 (with 168 pseudoknotted structures) contains 16 categories of pseudoknotted structures with at most 85% sequence similarities [2, 11]; and DK-pk16 contains 16 pseudoknotted structures with strong experimental support [12]. We note that all pseudoknotted structures are density-2 structures (i.e. are in class of structures handled by Iterative HFold) and there is no overlap between our four datasets. Table 2 summarizes the datasets used in this work.

**Table 2:** Summary of datasets used in this work.

Name	# of sequences	lengths	Reference
HK-PK	88	26–400	Andronescu et al. [3]
HK-PK-free	337	10–194	Andronescu et al. [3]
IP-pk168	168	21–137	Huang and Ali [11]
DK-pk16	16	34–377	Sperschneider et al. [12]

### 2.3 Accuracy

To evaluate prediction accuracy of the methods used in this work we use the harmonic mean of sensitivity and positive predictive value (PPV), commonly referred to as *F-measure*.

$$\begin{array}{c} \text{Sensitivity}=\frac{\text{Number}\;\text{of}\;\text{correctly}\;\text{predicted}\;\text{base}\;\text{pairs}}{\text{Number}\;\text{of}\;\text{base}\;\text{pairs}\;\text{in}\;\text{the}\;\text{reference}\;\text{structure}} \end{array}$$

$$\begin{array}{c} \text{PPV}=\frac{\text{Number}\;\text{of}\;\text{correctly}\;\text{predicted}\;\text{base}\;\text{pairs}}{\text{Number}\;\text{of}\;\text{predicted}\;\text{base}\;\text{pairs}} \end{array}$$

and

$$\begin{array}{c} F-\text{measure}=\frac{2\times \text{sensitivity}\times \text{PPV}}{\text{sensitivity}+\text{PPV}} \end{array}$$

Value of *F*-measure ranges between 0 and 1, with 0 indicating no common base pair with the reference structure and 1 indicating a perfect accuracy. When the denominators are 0, these values are set to 0.

### 2.4 Bootstrap percentile confidence intervals

We use bootstrap confidence intervals [13, 14] to assess the dependency of measured prediction accuracy of results of a method on a given set of RNA. We calculated 95% percentile confidence intervals of average *F*-measures for each method as explained in [5, 15]. We report the bootstrap 95% percentile confidence interval for average *F*-measure of each method, on our datasets. All calculations are performed using the “boot” package of the R statistics software environment [16].

### 2.5 Permutation test

We use a two-sided permutation test to assess the statistical significance of the observed performance differences between two methods. We follow the procedure explained in [5, 15]. All calculations are performed using the “permTS” method in the“perm” package of the R statistics software environment. We report a significance in prediction accuracy if the difference in *p*-values is less than 5%.

## 3 IPknot: A maximum-expected-accuracy-based method

Motivated by the finding of Mathews [17] that base pairs with high base pairing probabilities in the thermodynamic ensemble are more likely to exist in the known structure, methods based on maximum expected accuracy (MEA), predict the most probable structure [2, 18–20]. IPknot algorithm of Sato et al. [2] is an MEA-based method that employs the free energy parameters of HotKnots V2.0 [3], uses an approximation of base pairing probability distribution that considers pseudoknotted structures, and handles a wide class of pseudoknotted structures. In addition to RNA sequence, IPknot takes various input parameters; values of these parameters have a significant effect on performance of IPknot, and therefore must be chosen with care. To find the best performing combination of scoring model and weights we ran IPknot with all combinations on our datasets (we fixed complexity of structure at level 2, and did not use refinements). IPknot with NUPACK scoring model was not able to predict the structure for all sequences on our dataset. While IPknot performance with McCaskill and Contrafold scoring models was quite similar in almost all datasets, we found IPknot with Contrafold with *γ*_1_ = 4 and *γ*_2_ = 16 performing significantly better than the rest on HK-PK dataset. Therefor, in our work we compare IPknot with this setting (i.e. no refinement, scoring model = Contrafold, level = 2, and *γ*_1_ = 4 and *γ*_2_ = 16) with the rest of methods.

## 4 HotKnots: A heuristic method

Heuristic approaches for prediction of RNA secondary structure employ greedy methods to find the lowest energy structure with no guarantee of finding the minimum free energy structure. Such methods are mostly based on step-wise addition of structural features with the aim of minimizing the overall free energy, while the addition of features are not necessarily in any biologically important order [3, 21–26]. HotKnots method of Ren et al. [22] uses a similar technique but maintains a tree of candidate structures in which multiple partially formed structures are maintained and there are various possibilities in each case for addition of the next structural features. Given an RNA sequence, HotKnots first finds up to 20 lowest energy stems (from the set of all stems for the given RNA sequence), called *hotspots*. Then iteratively adds other non-overlapping low energy stems to these set of hotspots to produce up to 20 output structures. The original version of HotKnots employed energy model of Dirks and Pierce [10]. In the most updated version, i.e. HotKnots V2.0, Andronescu et al. [3] used machine learning techniques to estimate the energy parameters and improved HotKnots accuracy by 11% over its original energy model.

## 5 CCJ: An MFE-based method

The CCJ algorithm is based on the Minimum Free Energy (MFE) folding hypothesis, which states an RNA molecule folds into the structure with the minimum free energy. To predict the MFE secondary structure of pseudoknot-free secondary structures, a divide-and-conquer approach, based on dynamic programming can be used as follows. A pseudoknot-free secondary structure for a sequence is either closed by a base pair connecting the first and last base in the sequence, or can be broken down into two independent substructures. The total energy of a structure which is composed of two independent substructures is the sum of the energies of the loops of the substructures. The run-time of such algorithms is Θ(*n*^3^) for standard energy loop models [27], where *n* is the length of the sequence. Since MFE *pseudoknotted* secondary structure prediction is NP-hard [28–30], algorithms for MFE pseudoknotted secondary structure prediction trade off run-time complexity and the class of structures they “can handle”. We say that a given algorithm *can handle* a structure *R* if *R* is in the class of structures over which the algorithm optimizes.

Various polynomial time algorithms exist that handle pseudoknotted structures [10, 30–33]. All algorithms can handle H-type pseudoknots, and some can handle kissing hairpin structures when these do not have arbitrary nested substructures [32]. The most general MFE-based algorithm for prediction of pseudoknotted structures, Pknots, was proposed by Rivas and Eddy in 1999 [33]. Pknots is a complex dynamic programming algorithm that runs in Θ(*n*^6^) time, and Θ(*n*^4^) space.

**Fig 2:**
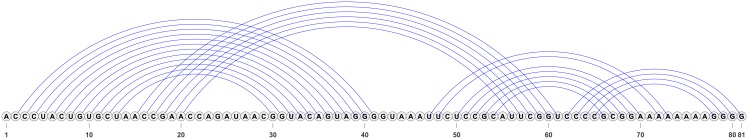
Arc diagram representation of a chain of four interleaved stems. Circles along the horizontal line represent bases from the 5’ (left) to the 3’ (right) ends. Arcs represent base pairs. Some arcs cross, thereby introducing pseudoknots.

**Fig 3:**
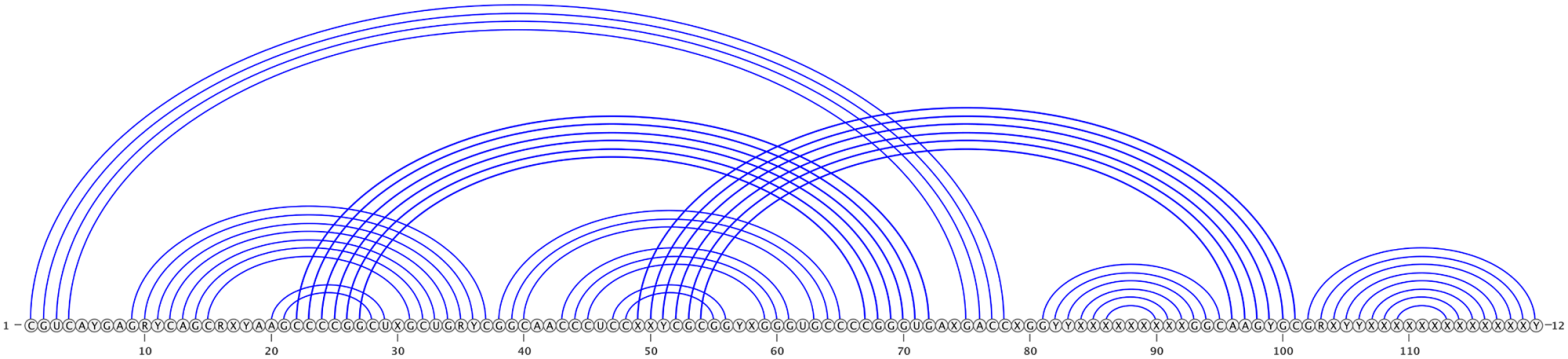
Arc diagram representation of SAM-IV riboswitch [34].

A recent algorithm put forward by Chen et al. [4], called CCJ, significantly expanded the class of structures that can be handled in *O*(*n*^5^) time and *O*(*n*^4^) space. For example, only CCJ can handle kissing hairpin structures with arbitrary nested substructures in *O*(*n*^5^) time, and this is a real limitation of previous methods given the biological importance of such structures. In addition, neither of the structures of Fig 2 nor Fig 3 can be handled by any of the algorithms with *O*(*n*^5^) running time, except CCJ. CCJ can also handle arbitrary nested substructures of these types.

**Fig 4:**
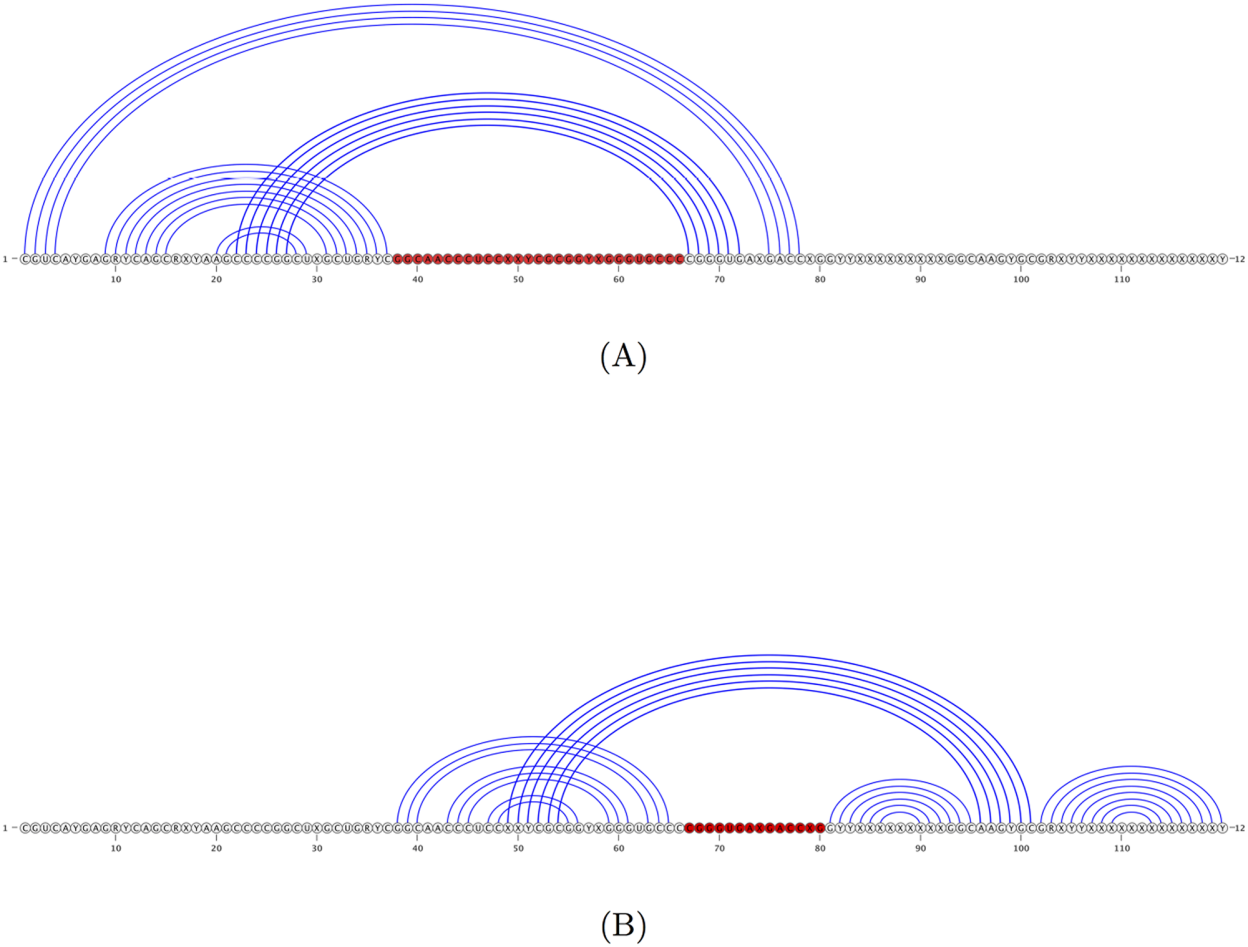
SAM-IV riboswitch of Fig 3, decomposed into two TGB structures. (A) represents a TGB structure with no band on the right; (B) represents a TGB structure. Red bases represent “gaps” in parsing the structure. Overlaying TGB structures (A) and (B) creates the SAM-IV riboswitch as a CCJ structure.

The CCJ algorithm describes a more general method of formulating the dynamic programming recurrences for prediction of pseudoknotted RNA secondary structures that cover “gapped regions”. It introduces two new ideas into the dynamic programming recurrences that improve the time complexity to *O*(*n*^5^): (i) a new class of structures called three-group-of-band (TGB) structures, with *at most* three groups of bands (where bands refer to consecutive base pairs that cross the same loop), and (ii) recurrences that handle TGB structures by transferring to the left, right, middle or the outer bands. CCJ algorithm can handle CCJ structures, that are constructed by overlaying two TGB structures. Fig 4 represent two TGB structures that overlay to create the CCJ structure of SAM-IV riboswitch presented in Fig 3.

The energy model used in the CCJ algorithm is a loop-based energy model, in which the energy of a secondary structure is calculated as sum of the energy of the structure’s loops. We used the DP09 energy model of HotKnots V2.0 [3], summarized in Table 1 in our implementation of the CCJ algorithm. The published version of the CCJ algorithm [4] uses a slightly more general model. There are few energy functions in the CCJ energy model that are not explicitly in the DP09 model. We have set values of these functions to 0, in order to make the models as similar as possible. We assume that any function specified in the energy model of the CCJ algorithm can be calculated in constant time, given sequence *S*.

## 6 Iterative HFold: A method based on the relaxed hierarchical folding hypothesis

Methods based on the hierarchical folding hypothesis have two potential advantages over MFE-based methods: (1) hierarchical folding hypothesis may model biological RNA folding just as well or better than does the MFE structure formation hypothesis, and (2) they are significantly more efficient in terms of time and space complexity when compared with the MFE-based methods that handle the same class of structures. The hierarchical folding hypothesis, which was first put forward by Tinoco and Bustamante [35], states that an RNA molecule first folds into a pseudoknot-free structure, then additional base pairs are added that may form pseudoknots with the first structure so as to lower the structure’s free energy. This hypothesis was first implemented in HFold, [7]. Given a pseudoknot-free structure as input, HFold predicts a (possibly) pseudoknotted structure that minimizes the free energy relative to the input structure. The class of structures handled by HFold, density-2 structures, is broad and HFold is significantly faster than MFE pseudoknotted secondary structure prediction methods (runs in *O*(*n*^3^) time and *O*(*n*^2^) space). However, HFold is unable to predict the correct structure if the input structure contains incorrect base pairs. While hierarchical folding hypothesis has been supported in the literature [36], there have been cases describing minor restructure of the originally formed pseudoknot-free structure upon formation of pseudoknot [37–39]. Motivated by the literature on the hierarchical folding hypothesis and to overcome some of the shortcomings of the MFE-based methods and those based on the hierarchical folding a new folding hypothesis, called “relaxed hierarchical folding” was recently described [5]. The first RNA pseudoknotted secondary structure prediction method based on this hypothesis, Iterative HFold, allows for minor modification in the input structure to minimize the overall free energy, while stays in the *O*(*n*^3^) time and *O*(*n*^2^) space complexity. Handling a general class of pseudoknotted structures (density-2 structures) and having the same time and space complexity as HFold (i.e. *O*(*n*^3^) time and *O*(*n*^2^) space), Iterative HFold improves significantly on its predecessor, achieving very good prediction performance while being one of the fastest pseudoknot prediction methods available. Iterative HFold uses HotKnots V2.0 DP09 parameter sets in its underlying energy model.

## 7 Results

By comparing four of the best methods for prediction of RNA pseudoknotted secondary structure (i.e. IPknot, a method based on maximum expected accuracy, HotKnots, a heuristic method, CCJ, a method based on MFE, and Iterative HFold, a method based on relaxed hierarchical folding hypothesis), while they all use the same energy model, we can evaluate (1) the merit of the hypothesis that RNA molecules fold into their MFE structures, and (2) significance of underlying prediction algorithm versus energy model. Therefore, we implemented optimized versions of Iterative HFold and CCJ algorithms to use HotKnots V2.0 DP09 energy model. Our implementations are available at our Github page. We start by describing the experimental settings for each of our computational experiments.

### 7.1 Input

Since Iterative HFold is the only method that requires an input structure, it is only fair if in comparison with other methods, no known structural information be provided as input structure to it. As demonstrated in [5], when there is no structural information available on a given RNA sequence, Iterative HFold performs best when provided with HotKnots hotspots as input structures. HotKnots hotspots are the 20 lowest energy pseudoknot-free stems produced in the first phase of HotKnots [3]. Here, we compare the average accuracy of Iterative HFold with HotKnots hotspots [3] as input structure. We choose the lowest free energy structure predicted by Iterative HFold for each set of hotspots as its final prediction given these hotspots.

Similarly we chose the lowest free energy structure predicted by HotKnots V2.0 (out of up to 20 structures predicted for each sequence) as the structure predicted by HotKnots to compare with other methods.

We ran IPknot with no refinement, scoring model = Contrafold, level = 2, and *γ*_1_ = 4 and *γ*_2_ = 16, as it yields the best results.

### 7.2 Platform

We ran all algorithms on Amazon Cloud (r4.8xlarge instance consisting of 32 Xeon E5-2686 Broadwell 2.3 GHz CPUs, and 244GB of DDR3 RAM).

### 7.3 Run time

On our dataset with sequences of 10 to 400 bases, IPknot took as little as 0.01 to 0.56 seconds to complete a run, compared to CCJ that took 0.05 to 576328.86 seconds (i.e. 6.67 days). Iterative HFold and HotKnots took 0.01 to 304.32 seconds (i.e. 5.072 minutes) and 0.10 to 2254.76 seconds (i.e. 37.58 minutes) respectively.

### 7.4 Accuracy comparison

**Fig 5:**
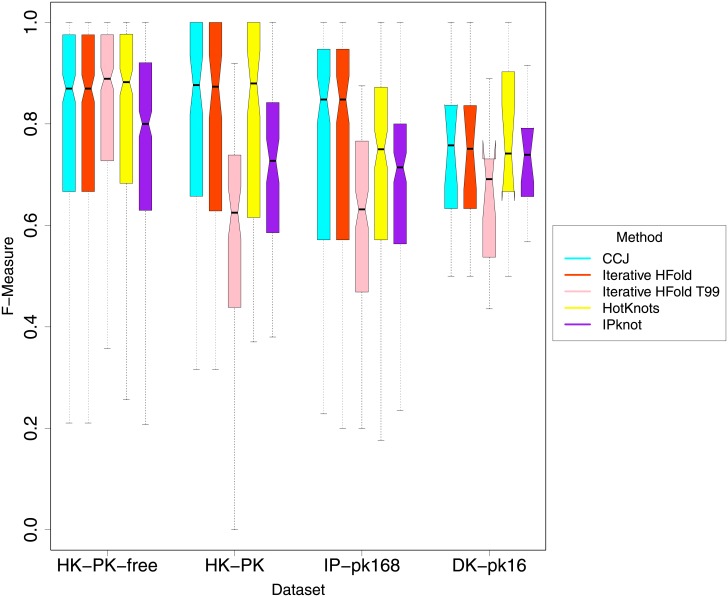
Comparison of bootstrap 95% percentile confidence intervals of average F-measure.

Fig 5 presents the bootstrap 95% percentile confidence intervals of average F-measure for all methods on all datasets. As shown in the figure and confirmed by our permutation tests the difference in accuracy of Iterative HFold, and CCJ on all datasets is not significant. On IP-pk168 dataset both Iterative HFold and CCJ perform significantly better than HotKnots V2.0 and IPknot. On HK-PK dataset both Iterative HFold and CCJ achieve significantly higher accuracies than IPknot, while their difference with HotKnots V2.0 is not significant. HotKnots V2.0 outperforms IPknot on both IP-pk168 and HK-PK datasets, but their accuracy difference on the rest of datasets is not significant.

**Fig 6:**
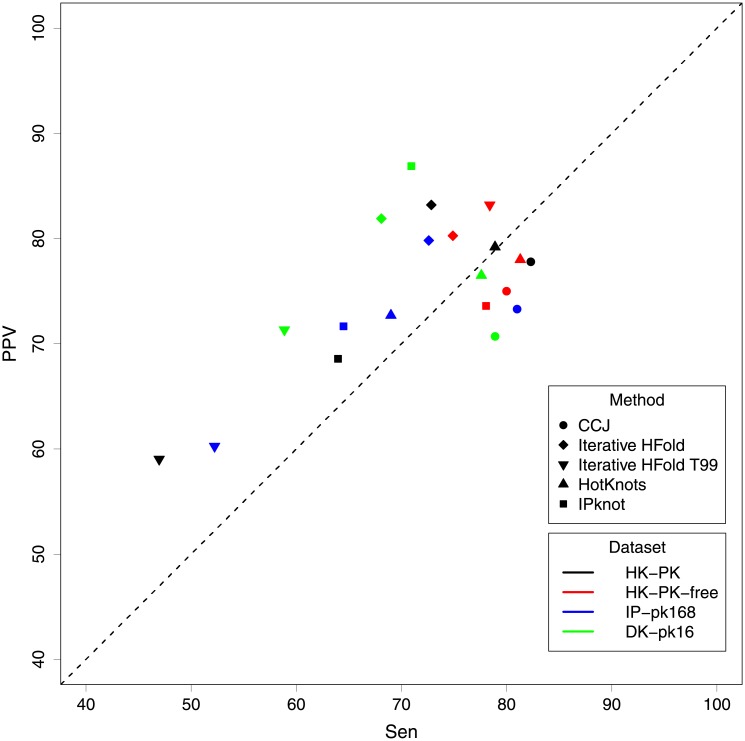
Comparison of sensitivity versus PPV of all methods on our four datasets. Points above the dashed line represent higher PPV value versus sensitivity while points below the dashed line represent higher sensitivity versus PPV value.

Fig 6 demonstrates sensitivity of all methods versus their PPV on all of our four datasets. As seen in Fig 6, Iterative HFold (i.e. diamond) has the highest PPV value in almost all our datasets, while CCJ (i.e. circle) has the highest sensitivity on all pseudoknotted datasets. HotKnots’ sensitivity and PPV values in almost all cases are close to the dashed line (i.e. almost equal); this might be due to the fact that the energy parameters were estimated based on HotKnots’ results. IPknot (i.e. square) in all pseudoknotted datasets has higher PPV than sensitivity while for pseudoknot-free dataset its sensitivity is higher than its PPV. It is interesting to note that while all other methods have similar performance on all our datasets, IPknot shows a lot of variations in its sensitivity versus PPV on different datasets.

To evaluate dependency of a method to its energy parameters, we ran Iterative HFold once more, this time with the standard Turner 99 parameters with Dirks and Pierce 2003 parameters for pseudoknotted structures, and refer to it by “Iterative HFold T99”. Values of this parameter set is presented in T99 column of Table 1. As expected, we see a significant degrade in the performance of Iterative HFold T99 compared to Iterative HFold. Our permutation test confirms the significance of the difference in all pseudoknotted datasets (see Fig 5). Also as seen in Fig 6 decline in both sensitivity and PPV of Iterative HFold T99 in all pseudoknotted datasets is clear. This emphasizes dependency of prediction accuracy of the method to its underlying energy parameters.

## 8 Discussion

In this section we evaluate merits of two biologically sound hypotheses on RNA folding (namely MFE and hierarchical folding hypotheses), by comparing performance of CCJ, HFold (a method base on strict hierarchical folding hypothesis, and one of the underlying methods of Iterative HFold) and Iterative HFold. More specifically we would like to see how a method that is purely based on MFE performs versus a method that strictly adheres to the hierarchical folding hypothesis and a method that is based on the “relaxed” hierarchical folding hypothesis. We did not find significant difference in accuracies of CCJ, HFold and Iterative HFold on any of our datasets. However, we observed that predicting the structure with the minimum free energy is not directly correlated with finding the structure with the highest accuracy among all methods. More specifically in the DK-pk16 dataset CCJ produces the lowest energy structure in all cases but only in one case produces the highest accuracy. This offers some support for the (relaxed) hierarchical folding hypothesis. We should note that this may be dependent also on the energy model used.

**Fig 7:**
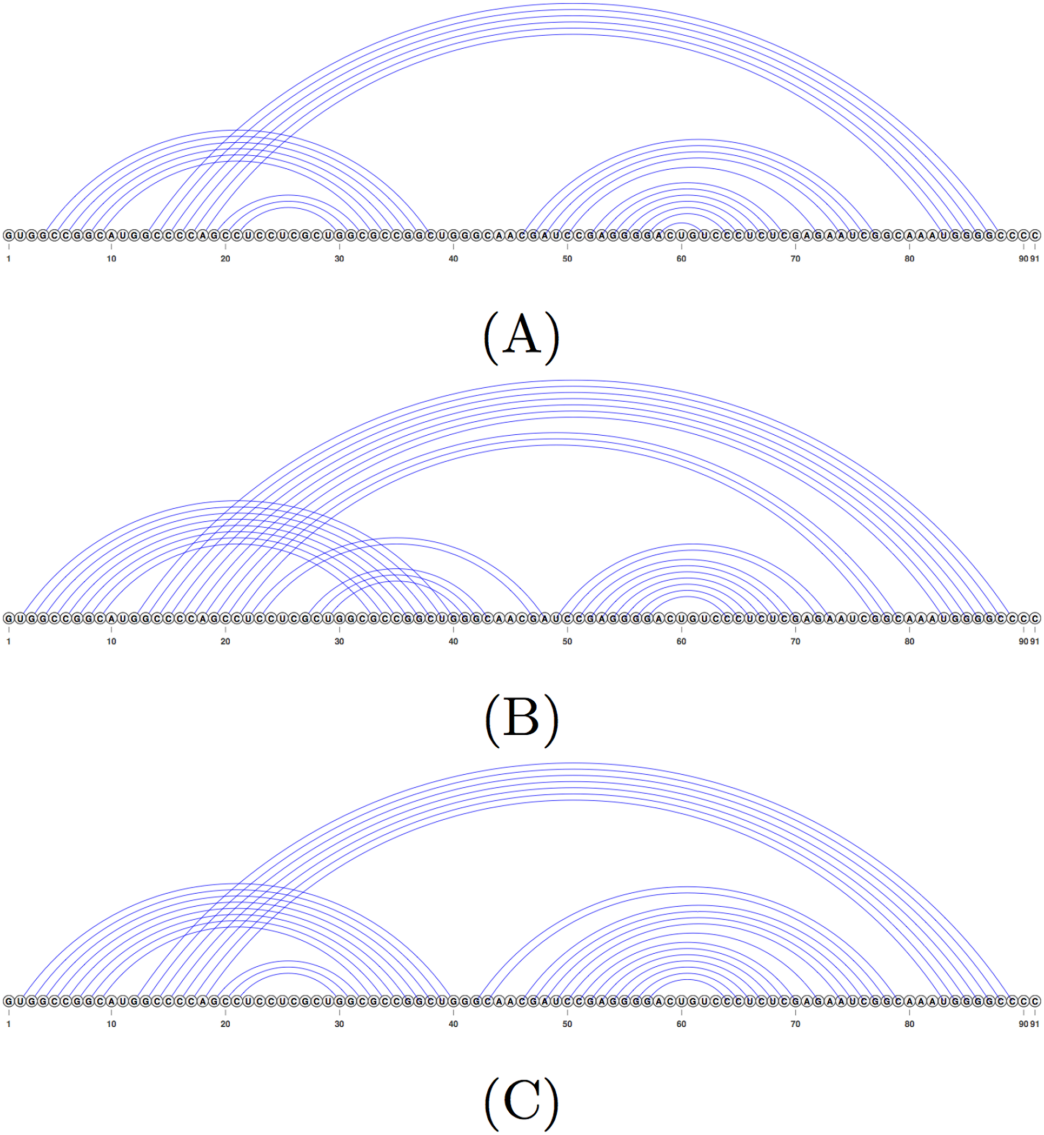
Example structure predicted by the CCJ algorithm vs. Iterative HFold. (A) Structure representation of the reference structure for sequence RFA_00632 of the HK-PK dataset. (B) The structure predicted by the CCJ algorithm given sequence RFA_00632. (C) The structure predicted by Iterative HFold for the same sequence. Both methods correctly identified the pseudoknotted stems, but CCJ added an extra pseudoknotted stem in place of the nested substructure in the reference structure.

Fig 7 shows an example of the structure produced by the CCJ algorithm, compared to the structure predicted by the Iterative HFold algorithm. The F-measure of the CCJ structure is 64.4% while Iterative HFold’s structure has an F-measure of 88.1%. As seen in Fig 7, both Iterative HFold and CCJ predicted the pseudoknotted stems in the reference structure. However, the structure predicted by CCJ missed the nested substructure and instead added another pseudoknotted stem.

**Fig 8:**
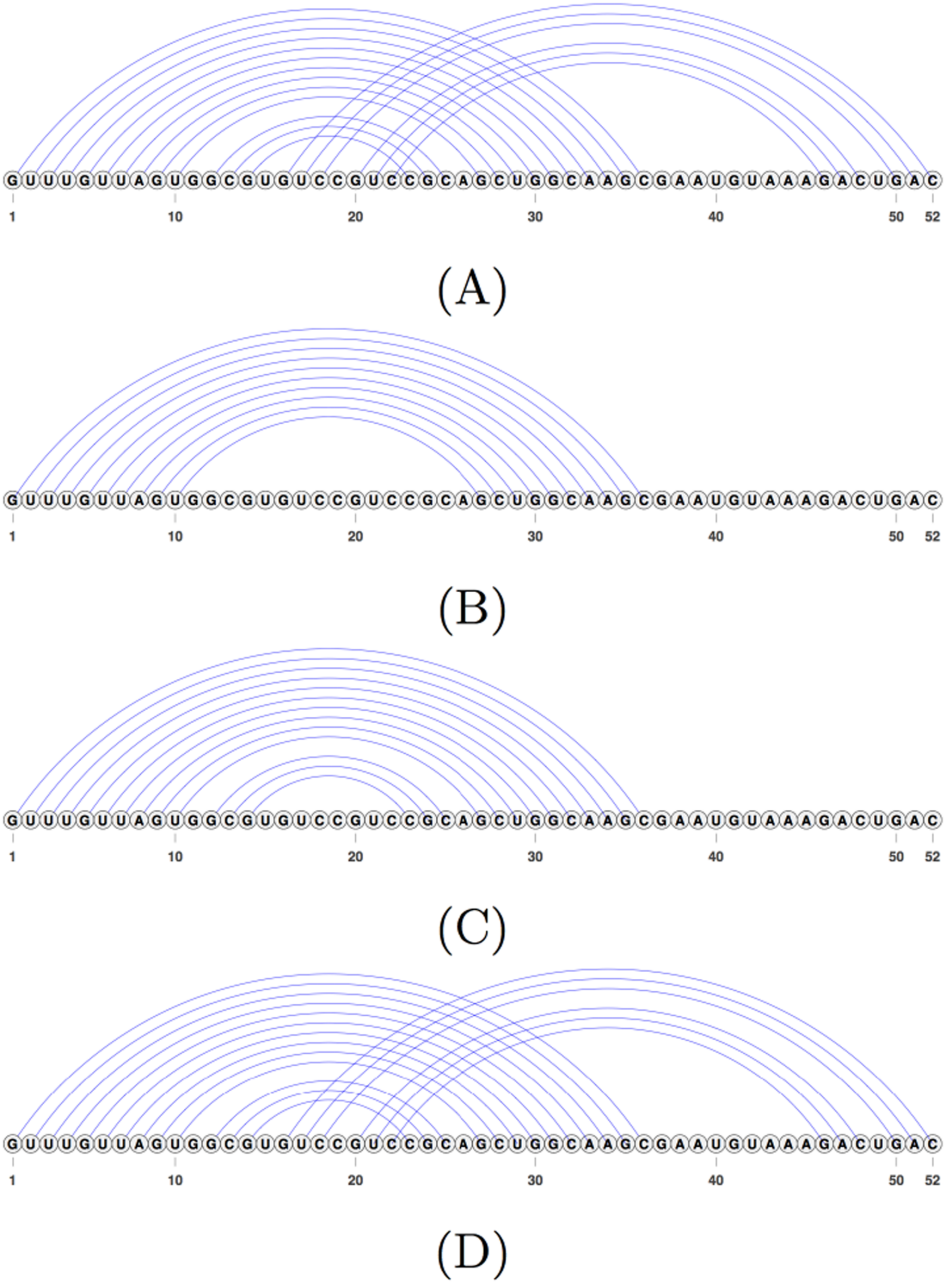
An example where Iterative HFold finds a structure with lower energy and higher accuracy than the structure predicted by HFold. (A) The reference structure for EC_PK4 of the HK-PK dataset. (B) The hotspot for EC_PK4 provided as input to both HFold and Iterative HFold resulting in their lowest energy structure prediction. (C) The structure predicted by HFold for EC_PK4 given the hotspot input (B). HFold is unable to find the pseudoknotted base pairs. (D) The structure predicted by Iterative HFold for EC_PK4 given the hotspot input (B). Iterative HFold successfully identified the pseudoknotted base pairs.

We also notice that by following the relaxed hierarchical folding hypothesis, hence allowing changes to the input structure, Iterative HFold more often finds structures with lower energy than those predicted by HFold in all of our datasets. However this does not result in significant improvement in prediction accuracy compared to HFold. Fig 8 shows an example of one such case, in which finding a lower energy structure results in predicting the reference structure. This also demonstrates HFold’s inability to add crossing base pairs to its input structure, and how Iterative HFold overcomes this limitation.

**Fig 9:**
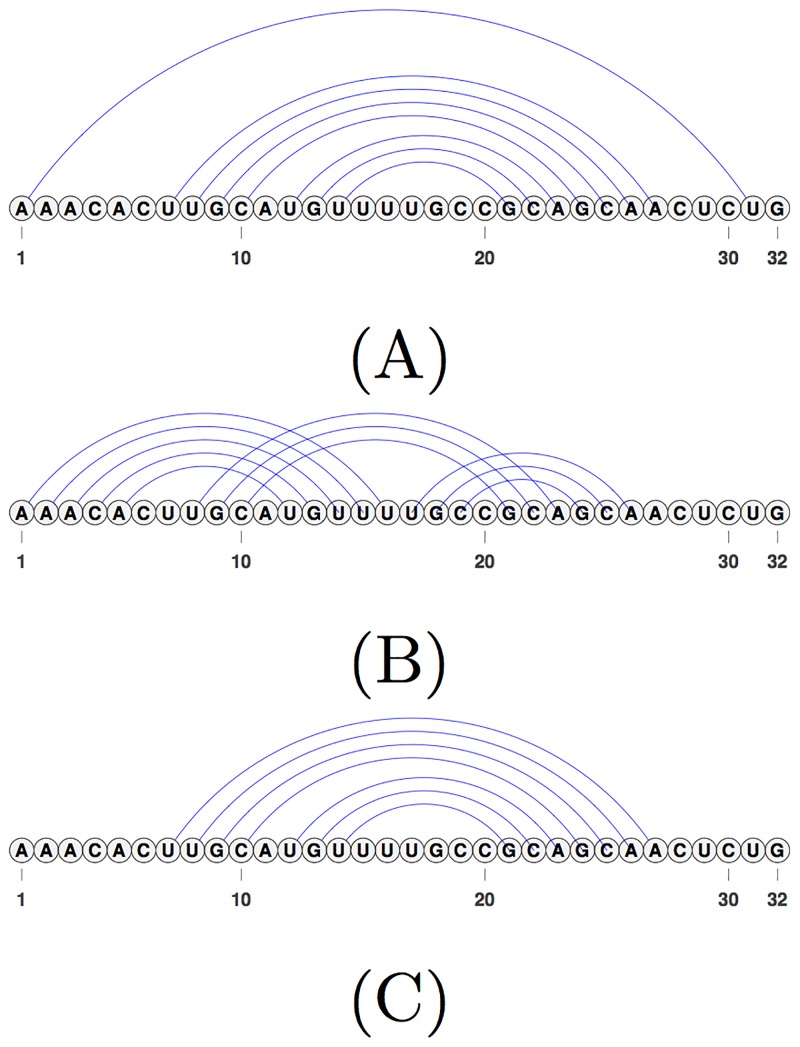
Example of a pseudoknot-free structure predicted as a pseudoknotted structure by CCJ. (A) The reference structure for CRW_00628 of the HK-PK-free dataset. (B) The structure predicted by the CCJ algorithm for sequence CRW_00628. CCJ identifies part of the reference structure (middle stem) but predicts the whole structure as pseudoknotted. (C) The structure predicted by Iterative HFold given the same sequence. Iterative HFold identifies all but one base pair of the reference structure.

In many cases, especially for longer RNA strands in the HK-PK-free dataset, the MFE structures predicted by the CCJ algorithm are pseudoknotted. This suggests that the energy parameters encourage addition of pseudoknots and so parameter estimation specifically for CCJ would be useful. Fig 9 shows an example of structures predicted by CCJ vs. Iterative HFold. In this example, the reference structure is a pseudoknot-free structure, consisting of single outermost base pair and a nested stem-loop structure. Due to high cost of the single base pair Iterative HFold does not predict it as part of its output structure, while predicting the rest of the structure correctly. CCJ on the other hand misses the pseudoknot-free structure completely and predicts a pseudoknotted structure (kissing hairpin).

Addition of pseudoknots in HFold’s predictions is the least among HFold, CCJ and Iterative HFold. This is mainly due to the fact that the hotspots input to HFold must also be part of the structure output by HFold, reducing HFold’s flexibility in adding pseudoknots (compared with CCJ and Iterative HFold). Iterative HFold’s predictions are mostly similar to those of HFold on the RNA sequences of the HK-PK-free dataset. However, in a few cases, especially in longer RNA strands Iterative HFold’s predictions are also pseudoknotted.

**Fig 10:**
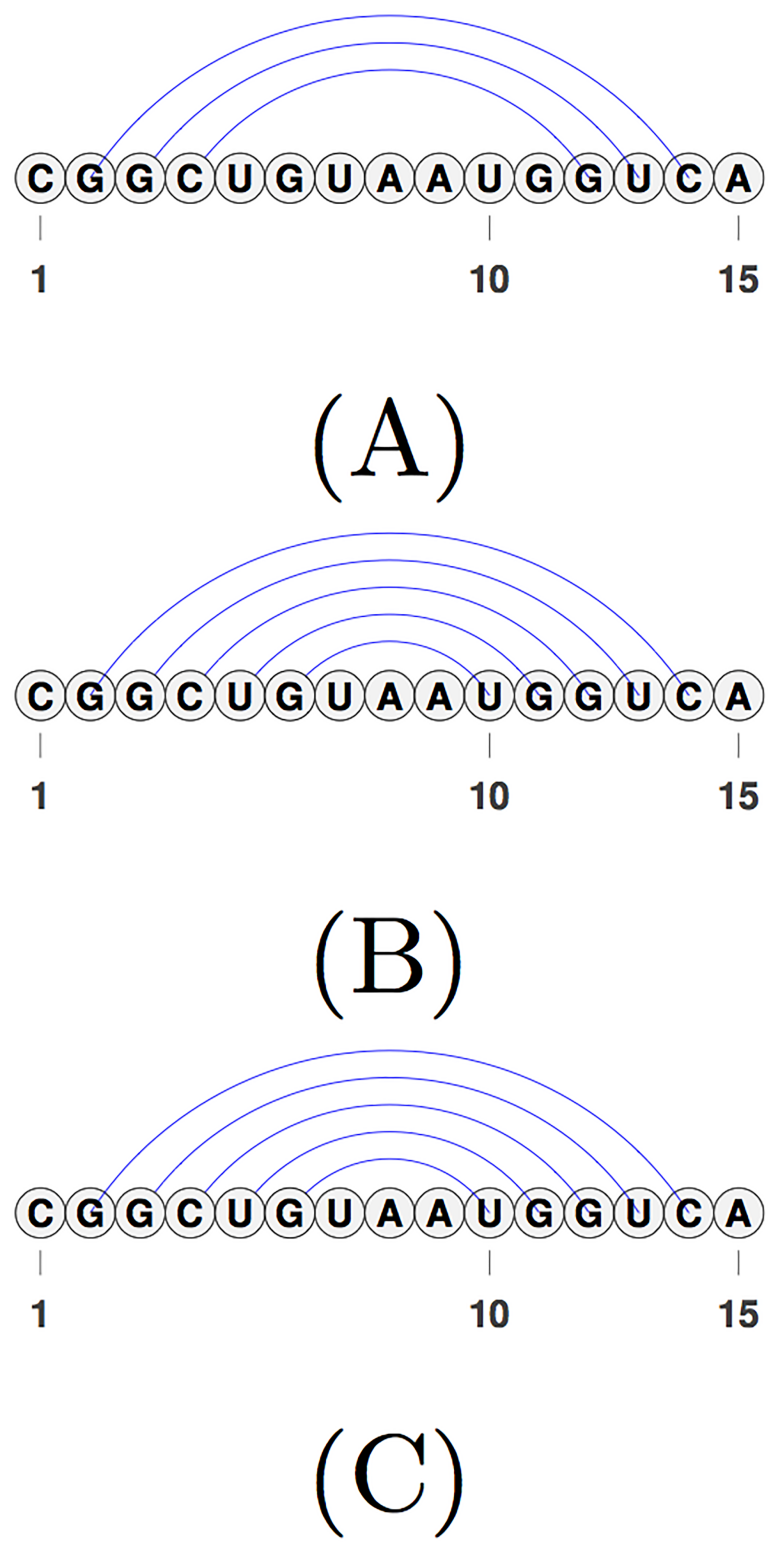
An example of overcrowded input structure provided to Iterative HFold. (A) The reference structure for SRP_00265 of the HK-PK-free dataset. (B) The hotspot input for SRP_00265 provided as input to Iterative HFold that resulted in prediction of the lowest energy structure. This input structure has more base pairs than the reference structure. (C) The structure predicted by Iterative HFold for SRP_00265 given hotspot (B). While the output structure predicted by Iterative HFold contains the base pairs of the reference structure, Iterative HFold is unable to remove base pairs of the overcrowded input structure.

On shorter RNA sequences, Iterative HFold is not able to modify the given structure, when the input structure is overcrowded with base pairs. Fig 10 shows an example in which input structure is overcrowded with base pairs, while the reference structure has a subset of base pairs in the input structure. In this example, removing identified base pairs causes increase in energy and therefore is not performed by Iterative HFold. This example is an illustration of either incorrect energy parameters or inaccurate reference structure.

**Fig 11:**
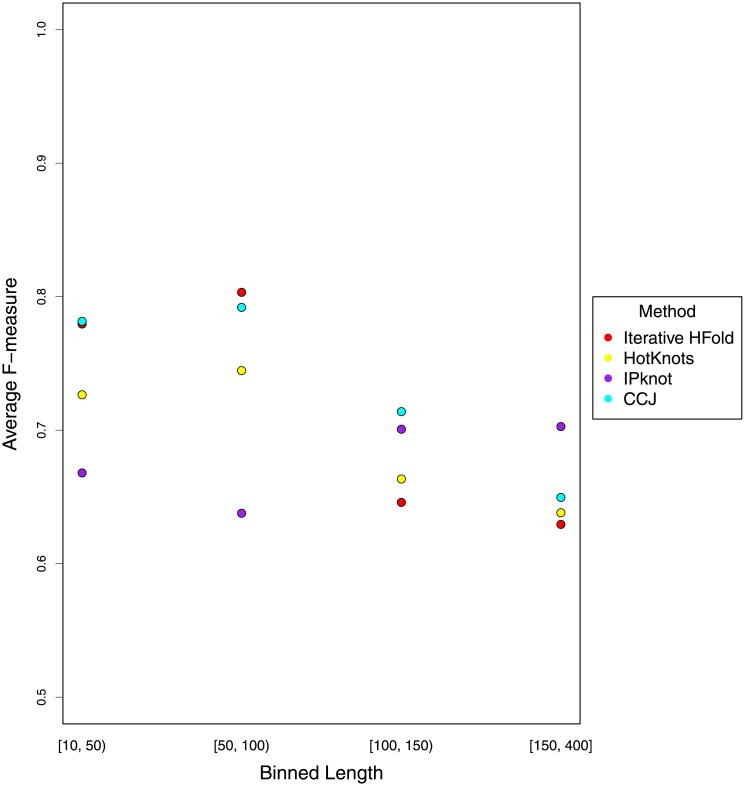
Comparison of average F-measure of each method based on sequence length on our pseudoknotted dataset.

To evaluate performance of the methods compared in this work based on sequence length, we combined all our pseudoknotted datasets, binned the sequences to [10, 50), [50, 100), [100, 150) and [150, 400], and reported average F-measure of each method in each case. Fig 11 summarizes this finding.

**Fig 12:**
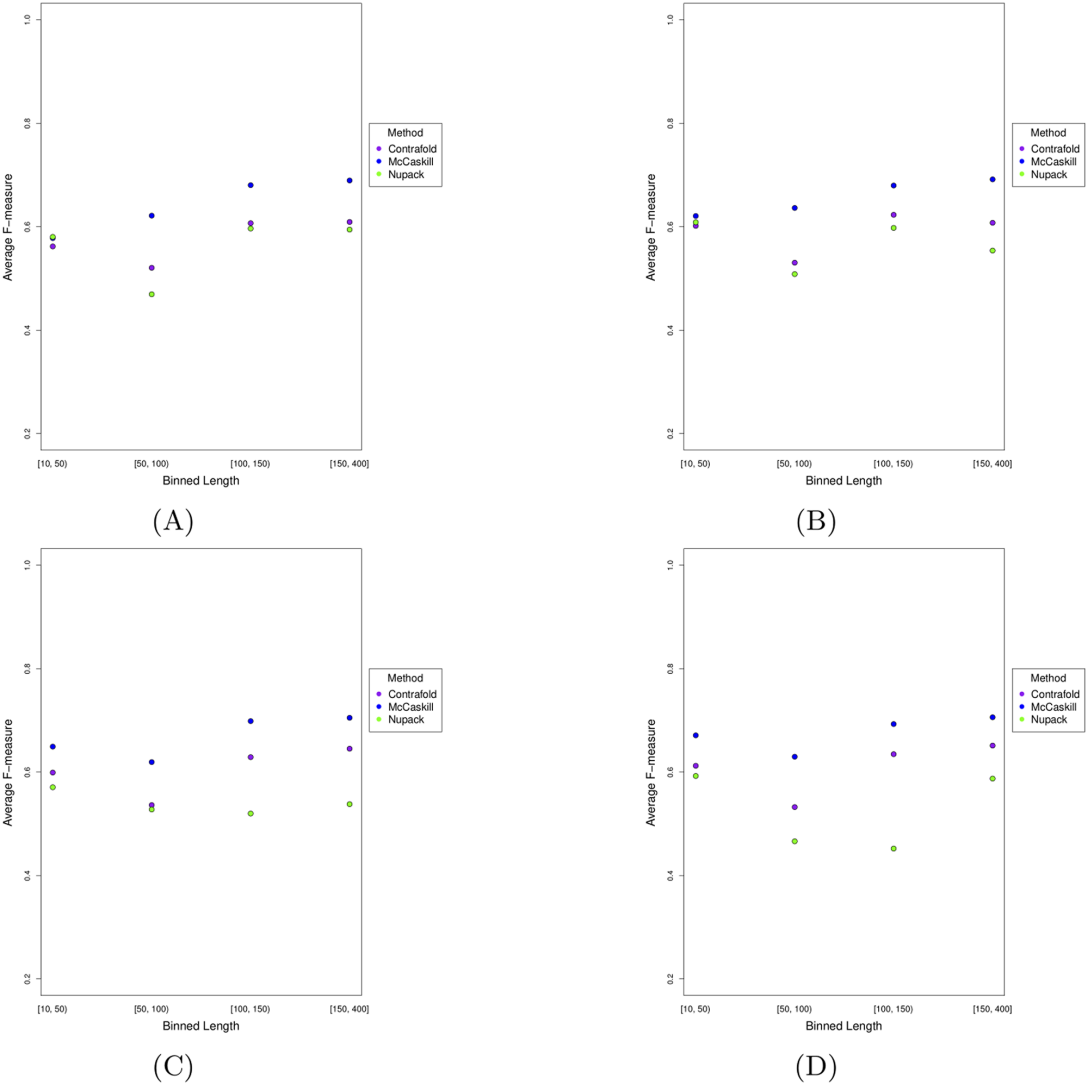
Comparison of average F-measure of IPknot on pseudoknotted structures with different scoring/energy models based on sequence length. Each figure shows a different weight combination: the weight combination represented in (A) is 2 and 4, in (B) is 2 and 16, in (C) is 4 and 8 and in (D) is 4 and 16.

As seen in Fig 11, in almost all compared methods (except IPknot), average F-measure declines by length. The methods accuracy increases up to length 100 and then decreases by length. IPknot is the only method that does not follow this trend. Dependency of prediction accuracy (i.e. average F-measure) on sequence length may be due to energy model parameters. To find out whether IPknot with a different energy model would follow the same trend seen in other methods, we ran IPknot with all combinations of energy model and weight values on our pseudoknotted dataset. As seen in Fig 12, regardless of the scoring model and weight combinations used for prediction of pseudoknotted structures, IPknot follows the same trend, and this trend is not similar to that of the rest of the methods compared in this work. IPknot with NUPACK scoring model did not produce results in all cases, so the number of sequences in each bin is not equal.

**Fig 13:**
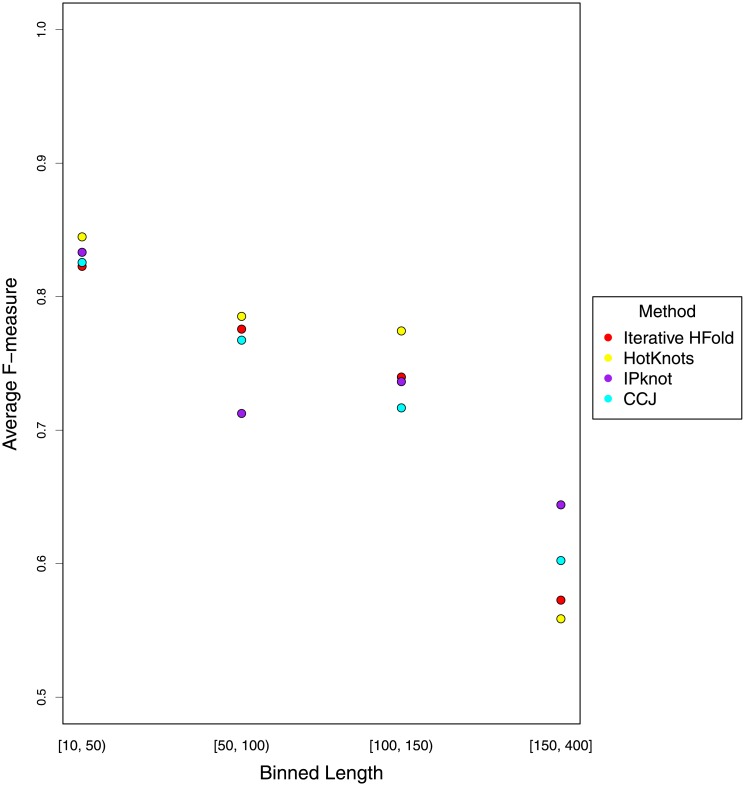
Comparison of average F-measure of each method based on sequence length on our pseudoknot-free dataset.

We further investigated dependency of prediction accuracy of all methods based on length on our pseudoknot-free dataset. As seen in Fig 13, accuracy decreases by length; this trend is consistent in all the methods compared in this work. This decrease in accuracy is in part due to addition of pseudoknotted base pairs.

## 9 Conclusion

In this work we thoroughly compared performance of four different methods for RNA pseudoknotted secondary structure prediction. All methods used similar energy models and two of them (Iterative HFold and CCJ) have biologically sound folding hypotheses. Our permutation test did not find significant difference in accuracy (computed as average F-measure) of Iterative HFold and CCJ with DP09 energy model, while the change in energy parameter values did make this difference significant. We also noticed a decline in average F-measure on length of the sequence in both methods; this is consistent with the finding of Lang et al. [40] for pseudoknot-free structure prediction. This decline in average F-measure might be due to inaccuracy of energy parameter values for long range interactions. We found that predicting the MFE structure does not necessarily translate to predicting the highest accuracy structure (i.e. structure with highest F-measure); this might be also due to inaccuracy of energy parameter values or due to our simplified assumption that RNA molecule folds in isolation while in nature it is guided (and restricted) by surrounding molecules. Looking closely at our findings we observed that

1. on long RNA sequences, CCJ tends to add more pseudoknotted base pairs;
2. on short RNA sequences, when the given input structure is overcrowded by base pairs, Iterative HFold is unable to modify the input structure to predict the reference structure;
3. CCJ has the highest sensitivity on all pseudoknotted datasets, while Iterative HFold has the highest PPV value; this indicates CCJ’s tendency to add pseudoknotted base pairs;
4. Iterative HFold in most cases predicts the overall “shape” of the structure correctly; F-measure alone is not a good measure for accurately predicting the overall shape of the structure.

Out of the four methods compared in this work, IPknot was the fastest and CCJ was the most expensive method to run. Therefore considering the differences in prediction accuracies, and time and memory requirements of the methods compared in this work, Iterative HFold with DP09 energy model might be the method of choice. However, we believe estimating the energy parameters specifically for each folding hypothesis might yield a different result as we find underlying energy model a significant contributor to prediction accuracy.
